# Contribution of CD4+ cells in the emotional alterations induced by endometriosis in mice

**DOI:** 10.3389/fnbeh.2022.946975

**Published:** 2022-10-12

**Authors:** Alejandra Escudero-Lara, David Cabañero, Rafael Maldonado

**Affiliations:** ^1^Laboratory of Neuropharmacology, Department of Experimental and Health Sciences, Universitat Pompeu Fabra, Barcelona, Spain; ^2^Hospital del Mar Medical Research Institute (IMIM), Barcelona, Spain

**Keywords:** endometriosis, anxiety, female, chronic pelvic pain, neuroinflammatory, CD4

## Abstract

Endometriosis is a disease defined by the presence of endometrial tissue in extrauterine locations. This chronic condition is frequently associated with pain and emotional disorders and has been related with altered immune function. However, the specific involvement of immune cells in pain and behavioral symptoms of endometriosis has not been yet elucidated. Here, we implement a mouse model of non-surgical endometriosis in which immunocompetent mice develop abdomino-pelvic hypersensitivity, cognitive deficits, anxiety and depressive-like behaviors. This behavioral phenotype correlates with expression of inflammatory markers in the brain, including the immune cell marker CD4. Depletion of CD4 + cells decreases the anxiety-like behavior of mice subjected to the endometriosis model, whereas abdomino-pelvic hypersensitivity, depressive-like behavior and cognitive deficits remain unaltered. The present data reveal the involvement of the immune response characterized by CD4 + white blood cells in the anxiety-like behavior induced by endometriosis in mice. This model, which recapitulates the symptoms of human endometriosis, may be a useful tool to study the immune mechanisms involved in pain and behavioral alterations associated to endometriosis.

## Introduction

Endometriosis is a chronic inflammatory disease that affects 1 in 10 women of reproductive age. It is defined by the presence of endometrial tissue outside the uterus and the main clinical manifestations are pelvic pain and infertility, often accompanied by anxiety, depression and reduced work productivity (Fourquet et al., 2011). There is a lack of correlation between the extent of the injury and the severity of the symptoms (Johnson et al., 2017) and the absence of obvious ectopic endometrial lesions does not eliminate the possibility of endometriosis. Pain-related symptomatology has been characterized in murine models of surgical (Escudero-Lara et al., 2020a) and non-surgical endometriosis (Fattori et al., 2020), although it is unknown whether the affective and cognitive alterations shown in surgical models are still reproducible in the absence of an initial surgery involving implantation of ectopic endometrium.

Endometriosis is associated with alterations in the immune system, and high levels of CD4 + lymphocytes and macrophages have been found in the peritoneal fluid of endometriosis patients (Becker et al., 1995; Gogacz et al., 2016). However, the possible contribution of this immunity to the development of endometriosis symptoms has not been yet investigated. This work characterizes the nociceptive, affective and cognitive manifestations developed in a murine model of non-surgical endometriosis conducted in immunocompetent mice. Brain neuroinflammatory changes are investigated and the participation of the immune response in endometriosis-related behavioral alterations is assessed through immunological targeting of CD4 + cells.

## Methods

### Animals

C57BL/6J 8-week-old female mice (Charles Rivers) were used in behavioral experiments. Female transgenic mice expressing GFP (C57BL/6 background) under the beta-actin promoter were provided by Prof. Muñoz-Cànoves (Universitat Pompeu Fabra, Spain). Mice were housed in cages of 4–5 mice with *ad libitum* access to water and food. Housing conditions were 21 ± 1°C and 55 ± 10% relative humidity in a controlled light/dark cycle. Mice were habituated to housing and handled for 1 week prior to experiments. All procedures were conducted in accordance with standard ethical guidelines (European Communities Directive 2010/63/EU and NIH Guide for Care and Use of Laboratory Animals, 8th Edition) and approved by the local ethical committees. Treatment groups were randomly assigned, and experiments were performed under blinded conditions.

### Endometriosis model

Mice received subcutaneous β-estradiol (3 μg/mouse, Sigma, cat.E8875) in corn oil (Sigma, cat.C8267) 7 days before induction of endometriosis. The phase of the estrous cycle on day 0 was assessed as previously described (Escudero-Lara et al., 2020b). Uterine horns of donors in diestrus were excised and opened longitudinally. The endometrium was separated by scraping with spatulas and suspended in vehicle (0.4 ml of saline with gentamicin 0.3 mg/ml). Endometrium obtained from one uterine horn was injected intraperitoneally in each recipient mice (endometriosis mice). Control mice received vehicle. This endometriosis model is similar to the one described by Fattori et al. (2020), except that in their protocol the complete uterine horns are homogenized in Hank’s balanced salt solution (HBSS) whereas in our method virtually only the endometrium is obtained and homogenized in saline containing gentamicin.

### Study design

**FIGURE 1:**
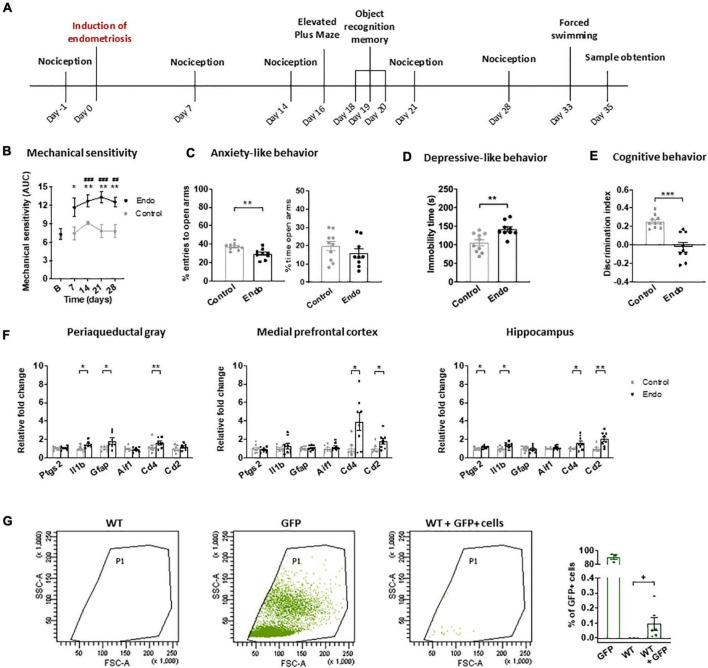
Behavioral and neuroinflammatory alterations in endometriosis mice. **(A)** Timeline of the behavioral experiment and sample collection. Endometriosis mice showed **(B)** persistent mechanical hypersensitivity in the abdomino-pelvic area after stimulation with von Frey filaments, **(C)** increased anxiety-like behavior in the elevated plus maze test, **(D)** increased depressive-like behavior in the forced swimming test and **(E)** cognitive impairment in the novel object recognition task. [**(F)** left panel] Endometriosis mice showed increased expression of interleukin 1-beta (*Il1b*), astrocyte marker GFAP (*Gfap*) and leucocyte marker CD4 (*Cd4*) in periaqueductal gray; [**(F)** central panel] overexpression of leucocyte markers CD4 (*Cd4*) and CD2 (*Cd2*) in medial prefrontal cortex, and [**(F)** right panel] high levels of cyclooxygenase 2 (*Ptgs2*), interleukin 1-beta (*Il1b*), CD4 (*Cd4*) and CD2 (*Cd2*) in hippocampus. Microglia marker IBA1 (Aif1) was unaltered. **(G)** Flow cytometry revealed 0.095 ± 0.042% GFP + cells in the peritoneum of WT mice 35 days after injecting GFP + endometrium. Error bars are mean ± SEM. **(B)** Mixed-model analysis **(C–E)** Student’s *t*-test. **(F,G)** Student’s *t* or Mann–Whitney *U* according to normality. ##*p* < 0.01, ###*p* < 0.001 vs baseline; *p < 0.05, **p < 0.01, ***p < 0.001 vs control; +*p* < 0.05 vs wild-type. Endo, endometriosis; AUC, area under the curve; GFP, green fluorescent protein; WT, wild-type.

Nociceptive, affective and cognitive behavior of endometriosis mice were evaluated in a first experiment (Figure 1A). Baseline mechanical sensitivity was assessed on day -1, and endometriosis or control injections were performed on day 0. Nociceptive responses were measured again on days 7, 14, 21, and 28. Anxiety-like, cognitive and depressive-like behavior were assessed on days 16, 20, and 33, respectively. On day 35, mice were euthanized for examination of the abdominal cavity.

In a second experiment, endometrium from GFP + mice was injected into wild-type littermates to determine whether endometrial cells could be found 35 days later. The presence of GFP + cells in the fluid obtained by peritoneal lavage was assessed by flow cytometry.

A third experiment was designed to study the specific role of CD4 + cells on the behavioral alterations associated with endometriosis (Figure 1B). Endometriosis mice were treated with 300 μg antiCD4 antibody GK1.5 (InVivoMAb, BioXCell, cat.BE0003-1) or vehicle (PBS) intraperitoneally on day 0 and boosted with additional doses of 100 μg antibody or vehicle on days 7, 14, 21, and 28. Behavioral measures were conducted as in the first experiment. The efficiency of CD4 + cell depletion was assessed by splenic flow cytometry on day 35.

### Behavioral evaluation

Mechanical sensitivity was quantified by measuring the responses to von Frey filament stimulation of abdomino-pelvic area as previously reported (Escudero-Lara et al., 2020a). Von Frey filaments (1.65, 2.36, 2.44, 2.83, 3.22, and 3.61 corresponding to 0.008, 0.02, 0.04, 0.07, 0.16, and 0.4 g; Bioseb, Pinellas Park, FL, USA) were applied in increasing order of force, 10 times each, for 1–2 s, with an inter-stimulus interval of 5–10 s. Abrupt retraction of abdomen, jumping and immediate licking of the site of application were considered positive responses. The area under the curve (AUC) was calculated by applying the linear trapezoidal rule to the plots representing the frequency of response versus the numbers of von Frey filaments, which represent the logarithm of the filament force expressed in mg ×10.

Anxiety-like behavior was analyzed with the elevated plus maze, a black plexiglas apparatus with 4 arms (29 cm long × 5 cm wide), 2 open and 2 closed, set in cross from a neutral square (5 cm × 5 cm) elevated 30 cm above the floor and indirectly illuminated from the top (40–50 lux in the open arms/4–6 lux in the close arms). Each mouse was placed in the central square facing the open arms and evaluated for 5 min. Percentage of entries and time spent in the open arms were measured.

Long-term object recognition memory was assessed with the V-maze (Panlab, Barcelona, Spain), as previously described (Escudero-Lara et al., 2020a). On the first day, mice were habituated for 9 min to the empty maze. The day after, mice were placed again in the maze for 9 min and 2 identical objects were presented at the ends of the arms of the maze. 24 h later, on the test day, one of the familiar objects was replaced with a novel one and each mouse was placed back in the maze for 9 min. The time spent exploring each object (novel and familiar) was recorded and a discrimination index was calculated as the difference between the time spent exploring the novel and the familiar object, divided by the total time exploring both objects. A threshold of 10 s of total interaction with the objects was set to discard low levels of general activity.

Depressive-like behavior was measured with the forced swimming test, as previously reported (Filliol et al., 2000). Mice were forced to swim for 6 min in a cylinder filled with water (23–25°C). Immobility duration was recorded for the last 4 min.

### Gene expression analysis

Periaqueductal gray, medial prefrontal cortices and hippocampi were obtained from untreated mice on day 35. RNA was isolated using Trizol^®^ (Invitrogen, cat.15596018) and reverse-transcribed with a High-Capacity cDNA Reverse-Transcription Kit (Applied Biosystems, cat.4368814). RT-PCR was performed with QuantStudio 12KFlex Real-Time PCR System (Applied Biosystems, cat.4471134) using SYBR Green PCR MasterMix (Roche, cat.04707516001). The following specific primers were used: 5′-CAGACAACATAAACTGCGCCTT-3′ (*Ptgs2* forward); 5′-GAT ACACCTCTCCACCAATGACC-3′ (*Ptgs2* reverse), 5′-GAAG TTGACGGACCCCAAAA-3′ (*Il1b* forward); 5′-TGATGTGC TGCTGCGAGATT -3′ (*Il1b* reverse), 5′-CAGAGGAGTGGT ATCGGTCTAAGTT-3′ (*Gfap* forward); 5′-CGATAGTCGTT AGCTTCGTGCTT-3′ (*Gfap* reverse), 5′-CCCCCAGCCAAG AAAGCTAT-3′ (*Aif1* forward), 5′-GCCCCACCGTGTGACAT C-3′ (*Aif1* reverse), 5′- CCCAGGTCTCGCTTCAGTTT -3′ (*Cd4* forward), 5′-GGGAGAGGTAGGTCCCATCA-3′ (*Cd4* reverse), 5′- ACCAACCTGAACGCACCATT -3′ (*Cd2* forward), 5′- CCAAGAGCACCAAGAGGAGT -3′ (*Cd2* reverse), 5′-CGTGAAAAGATGACCCAGATCA-3′ (*Actb* forward), 5′-CACAGCCTGGATGGCTACGT-3′ (*Actb* reverse). Data for each gene were analyzed by the 2^–ΔΔCt^ method after normalization to *Actb*.

### Flow cytometry analysis of peritoneal cells

Peritoneal lavages were performed with saline on day 35 to wild-type mice receiving GFP + endometrium. Samples from wild-type and GFP + mice served to establish gating conditions. Immunofluorescence was measured using BD™ LSR II flow cytometer (BD biosciences), and data were analyzed with FACSDiva™v6.2 software (BD biosciences).

### Confirmation of CD4 + cell depletion through splenic flow cytometry

Spleens from animals treated with vehicle or antiCD4 antibody were harvested on day 35. Tissues were smashed and filtered using 70-μm cell strainers (Falcon, cat.352350), and erythrocytes were lysed with RBC Lysis Buffer (Biolegend, cat.420301). The preparation was incubated with blocking antibody (1:100, anti-mouse CD16/32, BioLegend, cat.101301) 30 min on ice. Cells were then incubated 30 min on ice with DAPI (Sigma, cat.D9542), antiCD3 (1:100, CD3 Monoclonal (17A2), PE-Cyanine7, Invitrogen, cat.25-0032-82) and antiCD4 (1:100, FITC Rat AntiMouse CD4 Clone RM4-5, BD Biosciences, cat.553046). Immunofluorescence was measured using BD™LSR-II flow cytometer, and data were analyzed with FACSDiva™v6.2.

### Statistics

Statistical analyses were performed using IBM SPSSv24-25 software. Mixed-model analyses or repeated measures ANOVA followed by Bonferroni *post hoc* were used for repeated measures. Two-group comparisons were analyzed with *U* Mann–Whitney or Student’s *t*-test tests according to Shapiro–Wilk normality test and distribution data. Values outside the interval determined as mean ± 2 times standard deviation were excluded. Differences were significant when *p* < 0.05. A statistics and data source file containing the statistical analyses and the raw data is provided as Supplementary material.

## Results and discussion

The first aim was to investigate the nociceptive, affective and cognitive behavior of female mice receiving injected endometrial tissue (endometriosis mice) or vehicle (control) (Figure 1A). Endometriosis mice showed persistent mechanical hypersensitivity in the abdomino-pelvic area, whereas nociception of control mice remained unaltered (Figure 1B). Endometriosis mice also developed exacerbated anxiety-like behavior reflected in lower percentage of entries to the open arms of the elevated plus maze and a trend for lower percentage of time in the open arms (Figure 1C). These mice also showed increased depressive-like behavior revealed by longer immobility in the forced swimming test (Figure 1D). Accordingly, previous studies in models of surgical and non-surgical endometriosis described abdomino-pelvic hypersensitivity (Escudero-Lara et al., 2020a; Fattori et al., 2020), whereas affective-like disturbances were only previously described in surgical models (Li et al., 2018; Filho et al., 2019; Escudero-Lara et al., 2020a). The present non-surgical model uncovered depressive-like behavior in endometriosis mice, a phenotype that could not be evidenced before in our surgical model (Escudero-Lara et al., 2020a), possibly because of the impact of the sham surgery on the affective-like behavior of control mice. Endometriosis mice showed decreased discrimination indices in the novel object recognition task (Figure 1E), in agreement with our previous studies (Escudero-Lara et al., 2020a). Hence, this model reproduces the behavioral alterations shown in previous models of surgical endometriosis without the needs of inducing surgical injury. Furthermore, it allows the establishment of a differential depressive-like behavior in endometriosis mice, providing a proxy to investigate this comorbid disorder observed in clinical endometriosis.

To investigate whether endometriosis-driven behavioral alterations were accompanied by neuroinflammatory changes, mRNA expression of immune cell and inflammatory markers were determined in brain areas potentially involved in the nociceptive, emotional and cognitive impairments induced by endometriosis, i.e., periaqueductal gray, medial prefrontal cortices and hippocampi (Figure 1F). Endometriosis mice showed consistent higher expression of the immune cell marker CD4 (*Cd4*) in all the studied areas, whereas the T cell marker CD2 (*Cd2*) was significantly overexpressed only in cortices and hippocampi. Previous studies in neuropathic pain models suggested contribution of spinal cord CD4 + cells to pain-related behaviors (Labuz et al., 2009). In addition, expression of the astrocyte marker GFAP (*Gfap*) was increased in the periaqueductal gray of endometriosis mice, while expression of the microglia marker IBA1 (*Aif1*) remained unaltered in all the analyzed areas. Previous findings have described spinal astrocyte activation (Dodds et al., 2019) and spinal and supraspinal microgliosis (Chen et al., 2015; Liu et al., 2018) in different endometriosis models, however microglial markers were unaltered in our experimental conditions. While we did not detect any change at the mRNA transcript level, our data does not exclude possible significant changes in microglial cell activation or related protein expression. Endometriosis mice also enhanced interleukin 1-beta (*Il1b*) expression in periaqueductal gray and hippocampus, where cyclooxygenase-2 (*Ptgs2*) was also overexpressed. Accordingly, elevated cyclooxygenase-2 expression in the brain has been reported in a previous endometriosis model (Greaves et al., 2017). Overall, our data suggest a neuroinflammatory brain environment associated to the endometriosis phenotype. Specifically, our results reveal an immune response present in multiple brain areas, characterized by a consistent enhancement of the immune cell marker CD4, a glycoprotein mainly expressed by T-lymphocytes that is also found in other immune types such as monocyte/macrophages or dendritic cells (Sattentau and Weiss, 1988). Interestingly, CD4 was also described in brain neurons and additional central nervous system cells, where its expression and role merits further investigation (Omri et al., 1994).

Flow cytometry analysis of peritoneal lavage of wild-type mice receiving GFP + endometrium revealed significant presence of GFP + cells 35 days after injection (0.095 ± 0.042%, Figure 1G). Macroscopic endometriotic growths could not be detected in our experimental conditions, in contrast with previous results obtained in a non-surgical model of endometriosis (Fattori et al., 2020) where endometriotic cysts were found. Such a divergence could be due to methodological differences, since we scrapped the inner uterine layer to isolate endometrial tissue, while the former work described injection of complete uterine horns (Fattori et al., 2020). In line with our flow cytometry data, high presence of endometrial cells was described in the peritoneal fluid from endometriosis patients (Sharpe-Timms, 2005) and washings of the pelvic compartment were used to diagnose endometriosis (Cantley et al., 2018). Although, the extent of endometriotic lesions does not correlate with symptom severity in patients (Johnson et al., 2017) and microscopic endometriosis can cause high degrees of pain and associated symptoms, similar to other chronic pathologies such as osteoarthritis where the extent of the injury does not correlate with the painful symptomatology (Dieppe, 2004). Thus, mice subjected to this model of non-surgical endometriosis yielded significant numbers of endometriotic cells in the peritoneal lavage, reproducing clinical findings in which symptomatology correlates with abnormal detection of ectopic endometrial cells.

**FIGURE 2:**
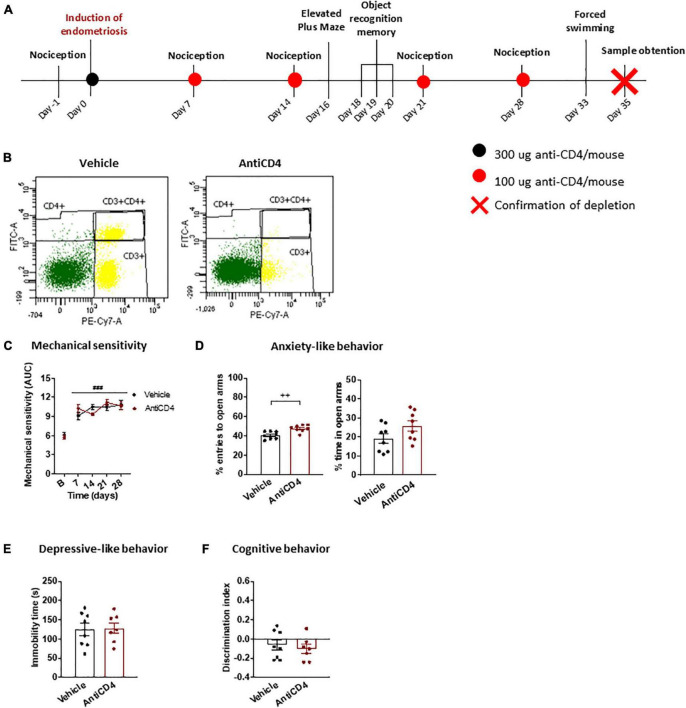
Behavioral effects of CD4 + cell depletion on endometriosis mice. **(A)** Timeline of the behavioral experiment and sample collection. **(B)** Splenic flow cytometry confirmed depletion of CD4 + cells. **(C)** Endometriosis mice treated with vehicle or antiCD4 antibody showed similar mechanical hypersensitivity after stimulation with von Frey filaments. **(D)** Depletion of CD4 + cells decreased anxiety-like behavior revealed by increased percentage of entries to the open arms of the elevated plus maze. **(E)** Depressive-like behavior in the forced swimming test was unaffected by CD4 + cell depletion. **(F)** Cognitive behavior in the object recognition task was similar regardless of CD4 + cells. Error bars are mean ± SEM. **(C)** Repeated measures ANOVA, **(D–F)** Student’s *t*-test. ###*p* < 0.01 vs baseline; ++ *p* < 0.01 vs vehicle. AUC, area under the curve; FITC-A, Fluorescein isothiocyanate A; PE-Cy7-A, R-phycoerythrin-Cyanine 7-A.

Our next objective was to investigate the participation of CD4 + cells in endometriosis-associated phenotypes using a CD4 + cell-depleting antibody. Depletion was confirmed at the end of the experiment through splenic flow cytometry (Figures 2A,B; depletion >98%). Endometriosis mice showed similar abdomino-pelvic hypersensitivity regardless of the antibody treatment (Figure 2C), and comparable to untreated endometriosis mice (Figure 1B), albeit the sensitization was slightly decreased likely because of the manipulations associated with the chronic treatments. In line with the similar sensitivity of CD4 treated or untreated mice, recent studies showed no differences in pain sensitivity between wild-type and T-cell deficient female mice subjected to chronic pain models (Rosen et al., 2017). Other authors evidenced pain-like responses preferentially mediated by white blood cells in females subjected to neuropathic injuries (Sorge et al., 2015), although antinociceptive responses have also been described in female and male mice subjected to other chronic pain models (Labuz et al., 2010; Laumet et al., 2019; Cabañero et al., 2020). Since mechanical sensitivity was unaffected by depletion of CD4 + cells, our results suggest that immune cells expressing this glycoprotein are not determinant for the hypersensitivity of this endometriosis model.

The antiCD4 treatment increased the percentage of entries to open arms in the elevated plus maze in endometriosis mice (Figure 2D), indicating inhibition of anxiety-like behavior induced by this condition. Interestingly, the reduction in anxiety-like behavior was particularly pronounced when compared to untreated endometriosis mice (Figure 1C). In agreement, it was described that the lack of CD4 + cells protects against stress-induced anxiety-like behaviors (Fan et al., 2019). We found this anxiolytic behavior in spite of the unaltered mechanical hypersensitivity, suggesting that increased anxiety-like behavior is not a direct consequence of enhanced pain sensitivity. This is in accordance with the increased anxiety-like behavior observed despite the alleviation of pain-like behaviors in a surgical model of endometriosis (Escudero-Lara et al., 2020a,^2021^). Altogether, the results suggest that CD4 + cells stimulated by the presence of ectopic endometrium contribute to a primary anxiogenic behavior in endometriosis mice. On the other hand, the time of immobility in the forced swimming test (Figure 2E) and the discrimination indices in the object recognition task (Figure 2F) were unchanged and similar to those of untreated endometriosis mice (Figures 1D,E), suggesting a lack of contribution of CD4 + cells to depressive-like behavior or cognitive impairment promoted by endometriosis. In agreement, wild-type and immune cell deficient male mice display similar depressive-like behaviors induced by inflammatory pain (Laumet et al., 2020). While it was also reported that immune cell deficiencies lead to cognitive dysfunction in naïve male mice (Kipnis et al., 2004; Ron-Harel et al., 2008), CD4 + cells did not play an apparent function on memory impairment in our model.

The present model of non-surgical endometriosis in mice suggests that a central inflammatory response may contribute to the affective symptomatology of endometriosis. However, the model has certain limitations including the lack of identification of the precise cell type expressing CD4, the absence of reproductible lesions that could allow the assessment of the evolution of the pathological condition, or a deeper characterization of the behavioral symptomatology that could identify whether the phase of the estrous cycle could be associated with the inflammatory response and/or the affective symptomatology. Further investigations in women with endometriosis may provide insight on the possible involvement of the inflammatory response on the emotional manifestations of this disease.

## Conclusions

Here, we show in a model of non-surgical endometriosis that ectopic endometrium elicits abdomino-pelvic hypersensitivity accompanied by anxiogenic and depressive-like responses and pronounced cognitive impairment. These alterations are similar to those observed in models of surgical endometriosis and do not require the presence of endometriotic cysts, resembling clinical findings in which symptom severity is unrelated with the size of the lesions. The behavioral phenotype of endometriosis mice correlates with increased expression of neuroinflammatory markers in brain areas related to nociceptive, emotional and cognitive processes, indicating that endometriosis may cause inflammation in these particular areas. CD4 + immune cells likely play a prominent role promoting an anxious phenotype, without modifying mechanical hypersensitivity, depressive-like behavior or cognitive deficits. The independence of anxiety-like behavior from these other behavioral alterations suggests a primary anxiogenic role of the immune response in individuals suffering from endometriosis. Further investigation is warranted to discriminate specific CD4 + cell types involved in the affective impairment associated with endometriosis.

## Data availability statement

The original contributions presented in this study are included in the article/Supplementary material, further inquiries can be directed to the corresponding authors.

## Ethics statement

This animal study was reviewed and approved by Comitè Ètic d’Experimentació Animal del PRBB (CEEA-PRBB).

## Author contributions

AE-L conducted all experiments. DC participated in initial experiments. AE-L, DC, and RM contributed to conception and design of the study. All authors contributed to manuscript revision, read, and approved the submitted version.
